# Clinical Factors Influencing Cosmetic Lip Reduction Recommendations: A Comparative Study of Orthodontists and Oral and Maxillofacial Surgeons

**DOI:** 10.7759/cureus.117454

**Published:** 2026-10-04

**Authors:** Varun Nambiar, Dhanalakshmi Varun, Rahul VC Tiwari

**Affiliations:** 1 Department of Oral and Maxillofacial Surgery, Dr. Nambiar’s Centre for Facial Cosmetic Surgery, Kochi, IND; 2 Department of Pedodontics and Preventive Dentistry, Dr. Nambiar’s Centre for Facial Cosmetic Surgery, Kochi, IND; 3 Department of Oral and Maxillofacial Surgery, RKDF Dental College and Research Centre, Bhopal, IND

**Keywords:** cosmetic lip reduction, lip morphology, oral and maxillofacial surgeons, orthodontists, treatment decision-making

## Abstract

Introduction: The esthetic lip morphology is influenced by soft-tissue characteristics, dental relationships, facial profile, and patient-related perceptions, which may contribute to differences in recommendations for cosmetic lip reduction. This study compared clinical factors and treatment recommendations between orthodontists and oral and maxillofacial surgeons using standardized written clinical scenarios.

Materials and methods: This cross-sectional comparative questionnaire-based study included 110 clinicians, comprising 55 (50.0%) orthodontists and 55 (50.0%) oral and maxillofacial surgeons. The participants evaluated six standardized clinical scenarios incorporating variations in lip fullness, lip prominence, dental protrusion, lip competence, strain, and facial characteristics. The questionnaire assessed the importance of clinical factors, scenario-based recommendations, treatment preferences, and clinical decision-making. Internal consistency was assessed using Cronbach's alpha. Between-group differences were evaluated using independent t-tests, Mann-Whitney U tests, chi-square tests, or Fisher's exact tests, as appropriate, with Holm-Bonferroni correction for multiple comparisons.

Results: Oral and maxillofacial surgeons assigned greater importance to lip-centric factors than orthodontists (p = 0.013), whereas orthodontists assigned greater importance to dental/skeletal and patient-related factors (both p < 0.001). Surgeons provided significantly higher recommendations for cosmetic lip reduction in five of the six standardized scenarios, whereas no significant difference was observed after the completion of orthodontic treatment (p = 0.113). Significant differences were also observed in treatment preferences, referral practices, multidisciplinary decision-making, objective facial analysis, orthodontic correction before surgery, and confidence in the recommendations. Questionnaire reliability was good, with Cronbach's α=0.85 for clinical factors, 0.88 for case assessment, and 0.79 for treatment preference.

Conclusion: Recommendations for cosmetic lip reduction differed between orthodontists and oral and maxillofacial surgeons, with the latter demonstrating a greater tendency to recommend reduction across several clinical scenarios. Orthodontists placed greater emphasis on dental and skeletal factors and orthodontic correction, whereas surgeons emphasized lip-related characteristics and objective facial assessments. These findings support individualized and multidisciplinary assessments before irreversible cosmetic lip interventions.

## Introduction

Facial attractiveness is influenced by the harmonious relationship among the lips, teeth, nose, chin, and surrounding soft tissues. The lips occupy a central position in facial esthetics and contribute substantially to facial proportions, profile harmony, smile appearance, and perceived age [1,2]. Variations in lip volume, projection, vermilion display, lip competence, and the relationship between the lips and dentoalveolar structures may therefore influence esthetic treatment planning [2,3]. Contemporary management of lip-related aesthetic concerns may involve minimally invasive or surgical approaches, including cosmetic lip lift procedures to modify vertical lip proportions and lip reduction (volume-reduction cheiloplasty) to address excessive lip volume [4]. Appropriate selection between these approaches requires consideration of the underlying facial and dental characteristics rather than the assessment of lip morphology in isolation.

Prominent or apparently excessive lips may result from increased soft tissue volume, dental protrusion, incisor proclination, skeletal relationships, or a combination of these factors. Orthodontic treatment may substantially modify lip position and facial profile when dentoalveolar protrusion contributes to lip prominence [5], whereas surgical intervention may be considered when excessive soft-tissue characteristics persist or are the principal esthetic concern [6,7]. Consequently, recommendations for cosmetic lip reduction may vary according to the clinician's specialty, clinical experience, interpretation of facial esthetics, and previous exposure to cosmetic lip procedures. Therefore, this study evaluated clinical decision-making using standardized written scenarios incorporating different combinations of lip fullness, lip prominence, lip competence, lip strain, dental protrusion, orthodontic characteristics, and facial profile features.

The primary objective of this study was to compare the recommendations for cosmetic lip reduction between orthodontists and oral and maxillofacial surgeons when evaluating standardized written clinical scenarios. The secondary objectives were to assess the perceived importance of clinical, facial, dental, soft-tissue, and patient-related factors influencing treatment recommendations; evaluate the influence of previous experience with cosmetic lip procedures; compare treatment preferences and broader clinical decision-making between the two professional groups; and assess differences in confidence, referral practices, and multidisciplinary involvement in decisions regarding cosmetic lip reduction.

## Materials and methods

Study design and setting

A cross-sectional, questionnaire-based analytical study was conducted to compare the clinical opinions and treatment decision-making of orthodontists and oral and maxillofacial surgeons regarding cosmetic lip reduction. The study was conducted in the Department of Oral and Maxillofacial Surgery, RKDF Dental College and Research Centre, Bhopal, India, during 2022. Data were collected over a period of four months, from June to September 2022. The study used a structured questionnaire incorporating standardized written clinical scenarios to ensure that all participants were presented with identical clinical information. The study was conducted in accordance with the ethical principles of the Declaration of Helsinki and received approval from the Institutional Ethical Committee of RKDF Dental College and Research Centre, Bhopal, India (Ref. No. RKDF/DC/2022/SS-010, dated May 09, 2022), prior to commencement of the study. Participant information and informed consent were incorporated into the questionnaire. Participants were informed about the purpose and procedures of the study, the voluntary nature of participation, the confidentiality of responses, and their right to withdraw without providing a reason. Responses were coded and analyzed in aggregate, and no unnecessary personally identifying information was collected.

Study population and eligibility criteria

A convenience sampling method was used to recruit eligible orthodontists and oral and maxillofacial surgeons. The study population comprised qualified orthodontists and oral and maxillofacial surgeons who were actively engaged in clinical practice. Participants were eligible if they had completed postgraduate training in their respective specialty, had at least one year of independent clinical experience following postgraduate qualification, were currently involved in clinical practice, and provided informed consent. Undergraduate and postgraduate students, clinicians who were not currently engaged in clinical practice, participants who submitted substantially incomplete questionnaires, and duplicate responses were excluded. The final study sample consisted of 110 clinicians, including 55 orthodontists and 55 oral and maxillofacial surgeons.

Questionnaire development and validation

A structured questionnaire was developed specifically for the study following a review of relevant literature concerning facial esthetics, lip morphology, orthodontic soft-tissue relationships, facial profile characteristics, and surgical management of excessive lip volume. The questionnaire was developed through a multidisciplinary process involving oral and maxillofacial surgeons, an orthodontist, and a pedodontist. The expert panel assessed the clinical relevance, clarity, comprehensiveness, and appropriateness of the proposed items, with emphasis on both soft-tissue and dentofacial factors that could influence recommendations for cosmetic lip reduction.

The questionnaire was designed to distinguish excessive lip volume from lip prominence attributable to dental or skeletal characteristics. The clinical factors included upper and lower lip fullness, overall lip volume, lip protrusion, lip incompetence, lip strain, vermilion show, lip asymmetry, gingival display, smile esthetics, maxillary and mandibular incisor proclination, bimaxillary dental protrusion, increased overjet, facial convexity, nasolabial angle, chin prominence, lower facial height, subjective cosmetic concern, and expected response to orthodontic treatment. Content validity was assessed using the item-level content validity index (I-CVI) and scale-level content validity index (S-CVI), with an I-CVI of ≥0.78 and an S-CVI/Ave of ≥0.90 considered acceptable [8,9]. Items considered unclear, redundant, or insufficiently relevant were revised before pilot testing. A pilot assessment was subsequently conducted among 20 eligible clinicians who were not included in the final study sample to assess clarity, comprehensibility, acceptability, completion time, and feasibility. Feedback from the pilot assessment was incorporated into the final questionnaire.

Construction of standardized clinical scenarios

The six standardized clinical scenarios were constructed to represent clinically distinct combinations of the principal factors considered relevant to recommendations for cosmetic lip reduction. The scenarios incorporated varying degrees of lip fullness and prominence together with dental protrusion, bimaxillary dental protrusion, incisor proclination, lip incompetence, lip strain, facial-profile characteristics, satisfactory dental alignment, and the persistence of lip fullness following orthodontic treatment. The scenarios were designed to distinguish situations in which apparent lip prominence could be related predominantly to soft-tissue characteristics from those in which a dentoalveolar component could contribute to the clinical presentation. They also included scenarios in which orthodontic treatment was expected to modify lip prominence and a scenario in which excessive lip fullness persisted after orthodontic correction. The scenarios were reviewed by the multidisciplinary expert panel for clinical relevance, clarity, internal consistency, and appropriateness before pilot testing. The panel review was intended to establish content appropriateness of the scenarios rather than to constitute formal validation of diagnostic or clinical case standards.

Questionnaire structure

The final questionnaire comprised four sections (Appendices). Section A collected demographic and professional characteristics, including age, sex, professional specialty, highest professional qualification, years of independent clinical experience, current practice setting, previous experience with cosmetic lip procedures, and the approximate number of patients with cosmetic lip concerns previously evaluated. Section B assessed the perceived importance of 20 clinical factors when deciding whether cosmetic lip reduction should be recommended. Each factor was rated using a 5-point Likert scale ranging from 1 (not important) to 5 (extremely important). For analysis, the items were grouped into four domains: lip-centric factors (B1-B8), facial and smile factors (B9-B10), dental and skeletal factors (B11-B18), and patient-related factors (B19-B20). Domain-level composite scores were calculated as the mean of the constituent item ratings for each participant.

Section C consisted of six standardized written clinical scenarios representing different combinations of lip morphology and dentofacial characteristics. The scenarios included variations in lip fullness, overall lip prominence, dental protrusion, bimaxillary protrusion, lip incompetence, lip strain, facial profile, satisfactory dental alignment, and persistence of lip fullness following orthodontic treatment. For each scenario, participants rated the severity of lip fullness, overall lip prominence, overall facial esthetics, and their recommendation for cosmetic lip reduction using 5-point scales. They also selected the principal clinical factor influencing their recommendation from a predefined list. The recommendation scale ranged from 1 (definitely not recommended) to 5 (definitely recommended).

Section D assessed treatment preferences and broader clinical decision-making. Participants were asked about the preferred initial treatment for patients with dental protrusion and prominent lips, whether cosmetic lip reduction should be deferred when orthodontic treatment was expected to substantially improve lip prominence, and whether cosmetic lip reduction should be recommended when excessive lip fullness persisted despite satisfactory dental alignment. Additional items assessed the perceived role of cosmetic lip reduction, the importance of patient perception and objective facial analysis, the importance of orthodontic correction before surgery, confidence in making recommendations, referral for another specialist opinion, and preferred specialist involvement in treatment planning.

The questionnaire was designed to distinguish excessive lip volume from lip prominence attributable to dental or skeletal characteristics. The 20 clinical factors were grouped a priori into four conceptual domains according to their primary clinical construct: lip-centric factors (B1-B8), facial and smile factors (B9-B10), dental and skeletal factors (B11-B18), and patient-related factors (B19-B20). The lip-centric domain represented direct characteristics of lip morphology and function; the facial/smile domain represented broader facial and smile aesthetics; the dental/skeletal domain represented dentoalveolar and skeletal characteristics that may contribute to lip prominence; and the patient-related domain represented subjective cosmetic concern and the anticipated response to orthodontic treatment. The grouping was informed by the literature reviewed during questionnaire development and refined through multidisciplinary expert input. It was used as a conceptual and analytical framework for the present study and was not intended to represent a previously validated classification system.

Because all six scenarios assessed the same underlying response construct, recommendation for cosmetic lip reduction, using an identical 5-point response scale, an overall recommendation score was calculated as the mean recommendation rating across the six scenarios. This score was intended as a summary measure of each participant's general tendency to recommend cosmetic lip reduction across the range of standardized clinical situations represented in Section C, rather than as an indication that the scenarios were clinically equivalent. Scenario-specific recommendation scores were analyzed separately to preserve the clinical distinctions between cases.

Outcome measures and scoring

The primary outcome was the recommendation for cosmetic lip reduction across the six standardized clinical scenarios, with the principal comparison being between orthodontists and oral and maxillofacial surgeons. Recommendation scores of 4 or 5 were classified as a recommendation for cosmetic lip reduction, scores of 1 or 2 as no recommendation, and a score of 3 as uncertain. The overall recommendation score was calculated as the mean recommendation rating across the six scenarios, with higher scores indicating a greater tendency to recommend cosmetic lip reduction. Secondary outcomes included the perceived importance of individual clinical factors and their domain-level scores, scenario-based assessments, principal factors influencing recommendations, treatment preferences, confidence in decision-making, previous experience with cosmetic lip procedures, and referral or multidisciplinary decision-making preferences. Internal consistency was assessed separately for the multi-item substantive sections of the questionnaire using Cronbach’s alpha. Section B comprised 20 clinical-factor items (B1-B20), Section C comprised the six standardized clinical scenario assessments, and Section D comprised 10 treatment-preference and clinical decision-making items. Section A, which consisted of demographic and professional characteristics, was not included in the reliability analysis.

Sample size calculation

The sample size was determined a priori based on the primary objective of comparing recommendations for cosmetic lip reduction between orthodontists and oral and maxillofacial surgeons. As no previous study directly addressing this specific comparison was identified, an effect size could not be derived from a directly comparable study. An a priori power analysis was therefore performed using G*Power, version 3.1.9.7 (Heinrich-Heine-Universität Düsseldorf, Düsseldorf, Germany), using a two-group comparison framework. A two-sided significance level of 0.05 and statistical power of 80% were assumed. The required sample was approximately 50 participants per group, and therefore a minimum total sample of approximately 100 participants was targeted. The final sample comprised 110 participants, with 55 orthodontists and 55 oral and maxillofacial surgeons.

Statistical analysis

Data were analyzed using Python (Python Software Foundation, Wilmington, DE, USA), with the SciPy, NumPy, and pandas libraries. Continuous and composite variables were assessed for normality using the Shapiro-Wilk test. Individual Likert-scale items and scenario-based ordinal ratings were analyzed using the Mann-Whitney U test for between-group comparisons. Composite/domain scores, calculated as the mean of constituent items, were assessed for normality and compared using the independent-samples t-test, with Welch's correction where appropriate, when normally distributed, or the Mann-Whitney U test when the normality assumption was not met. The overall recommendation score, calculated as the mean recommendation rating across the six standardized scenarios, was analyzed as a composite score using the same distribution-based approach. Categorical outcomes, including principal clinical factors, treatment preferences, referral patterns, and preferred specialist involvement, were compared using the chi-square test of independence or Fisher's exact test, as appropriate. Effect sizes were reported according to the analysis performed. The Holm-Bonferroni correction was applied for multiple item-level comparisons. Statistical significance was considered at a two-sided p < 0.05.

## Results

A total of 110 participants were included, comprising 55 (50.0%) orthodontists and 55 (50.0%) oral and maxillofacial surgeons. Age and years of professional experience did not differ significantly between the groups. Significant differences were observed in sex distribution (p = 0.020), highest qualification (p = 0.037), practice setting (p = 0.019), previous cosmetic lip experience (p = 0.032), and the number of patients with lip concerns previously evaluated (p = 0.002). Previous cosmetic lip experience was reported by 16 (29.1%) orthodontists and 27 (49.1%) oral and maxillofacial surgeons (Table 1).

**Table 1:** Demographic and professional characteristics of participants. Data are presented as frequency and percentage for categories; continuous data are presented as mean ± standard deviation. Between-group comparisons were performed using the independent t-test for normally distributed continuous variables and the chi-square (χ²) test for categorical variables. OR is reported for 2×2 comparisons, with the second-listed category used as the reference category; Cramer's V is reported for larger contingency tables. *p < 0.05 is considered statistically significant. MDS: Master of Dental Surgery, DNB: Diplomate of National Board, OR: odds ratio, CI: confidence interval.

Variable		Orthodontists (n = 55)	Oral surgeons (n = 55)	Test statistic	p-value	Effect size
Age, mean ± SD	Years	39.2 ± 6.8	40.0 ± 7.5	t = -0.64	0.522	d = -0.12
Sex, n (%)	Female	29 (52.7%)	17 (30.9%)	χ² = 5.38, df = 1	0.020*	OR = 0.40
	Male	26 (47.3%)	38 (69.1%)			
Highest qualification, n (%)	MDS	39 (70.9%)	32 (58.2%)	χ² = 6.59, df = 2	0.037*	Cramer's V = 0.24
	DNB	10 (18.2%)	21 (38.2%)			
	Other	6 (10.9%)	2 (3.6%)			
Years of experience, n (%)	1-5 yrs	19 (34.5%)	14 (25.5%)	χ² = 1.14, df = 2	0.566	Cramer's V = 0.10
	6-10 yrs	15 (27.3%)	16 (29.1%)			
	>10 yrs	21 (38.2%)	25 (45.4%)			
Practice setting, n (%)	Academic	19 (34.6%)	10 (18.2%)	χ² = 9.92, df = 3	0.019*	Cramer's V = 0.30
	Hospital	7 (12.7%)	18 (32.7%)			
	Private	28 (50.9%)	23 (41.8%)			
	Other	1 (1.8%)	4 (7.3%)			
Prior cosmetic-lip experience, n (%)	Yes	16 (29.1%)	27 (49.1%)	χ² = 4.62, df = 1	0.032*	OR = 0.43
	No	39 (70.9%)	28 (50.9%)			
Patients with lip concerns evaluated, n (%)	None	9 (16.4%)	2 (3.6%)	χ² = 17.03, df = 4	0.002*	Cramer's V = 0.39
	1-10	17 (30.9%)	11 (20.0%)			
	11-25	18 (32.7%)	11 (20.0%)			
	26-50	7 (12.7%)	18 (32.7%)			
	>50	4 (7.3%)	13 (23.7%)			

Significant between-group differences were observed in the importance assigned to lip-centric, dental/skeletal, and patient-related factors. Oral and maxillofacial surgeons assigned greater importance to lip-centric factors (3.62 ± 0.54 vs. 3.38 ± 0.45; p = 0.013), whereas orthodontists placed greater emphasis on dental/skeletal (3.85 ± 0.38 vs. 3.26 ± 0.58; p < 0.001) and patient-related factors (p < 0.001). No significant difference was observed between the facial and smile factors (p = 0.489) (Table 2).

**Table 2:** Comparison of domain-level composite importance scores for clinical factors in section B. Data are presented as mean ± standard deviation for normally distributed composite scores and median (IQR) for non-normally distributed scores. Composite scores represent the mean of the constituent item ratings on a 1–5 scale; normally distributed scores were compared using the independent-samples t-test, with Welch's correction where appropriate; non-normally distributed scores were compared using the Mann–Whitney U test; *p < 0.05 considered statistically significant.

Domain	Orthodontists (n = 55)	Oral surgeons (n = 55)	Test used	Test statistic	p-value	Effect size	95% CI
Lip-centric factors (B1-B8), mean ± SD	3.38 ± 0.45	3.62 ± 0.54	Independent t-test	t = -2.53	0.013*	d = -0.48	-0.43, -0.05
Facial/smile factors (B9-B10), median (IQR)	3.50 (3.00–3.75)	3.50 (3.00–4.00)	Mann-Whitney U	U = 1625	0.489	r = 0.06	-0.50, 0.50
Dental/skeletal factors (B11-B18), mean ± SD	3.85 ± 0.38	3.26 ± 0.58	Independent t-test (Welch)	t = 6.24	< 0.001*	d = 1.19	0.40, 0.77
Patient-related factors (B19-B20), median (IQR)	4.00 (3.75–4.50)	3.50 (3.00–4.00)	Mann-Whitney U	U = 2083	< 0.001*	r = 0.33	0.00, 0.50

Across the six standardized clinical scenarios, recommendations for cosmetic lip reduction were significantly higher among oral and maxillofacial surgeons in five scenarios (C1-C5; all Holm-adjusted p < 0.001), with effect sizes ranging from r = 0.38 to 0.46. No significant difference was observed for persistent lip fullness after the completion of orthodontic treatment (C6; p = 0.113) (Table 3).

**Table 3:** Comparison of ratings across the standardized clinical scenarios in section C. Data are presented as median (interquartile range as IQR). Severity, prominence, esthetics, and recommendation were rated on a 1–5 scale. Between-group comparisons were performed using the Mann–Whitney U test; effect size is reported as r. Holm-adjusted p-values were calculated across the 24 comparisons (six scenarios × four ratings) to control the family-wise error rate; *p < 0.05 was considered statistically significant after Holm adjustment.

Clinical scenario	Rating	Orthodontist, median (IQR)	Oral surgeon, median (IQR)	U value	Z value	p-value (nominal)	p-value (Holm-adjusted)	Effect size (r)	95% CI median diff.
C1: Mild lip fullness, no dental protrusion	Severity	2 (1–2)	2 (2–2)	1144	-2.21	0.014*	0.254	0.21	0.00, 0.00
	Prominence	2 (1–2)	2 (1–3)	1246	-1.59	0.090	1.000	0.15	-1.00, 0.00
	Esthetics	4 (3–4)	4 (3–4)	1838	1.95	0.036*	0.583	0.19	0.00, 1.00
	Recommendation	2 (1–2)	2 (2–3)	854	-3.94	< 0.001*	< 0.001*	0.38	-1.00, 0.00
C2: Moderate lip fullness, satisfactory occlusion	Severity	3 (2–3)	3 (3–4)	1316	-1.17	0.204	1.000	0.11	0.00, 0.00
	Prominence	3 (3–4)	3 (3–3)	1612	0.59	0.505	1.000	0.06	0.00, 0.00
	Esthetics	3 (2–4)	3 (2–4)	1654	0.85	0.375	1.000	0.08	0.00, 1.00
	Recommendation	2 (2–3)	4 (3–4)	709	-4.80	< 0.001*	< 0.001*	0.46	-2.00, 0.00
C3: Prominent lips with bimaxillary protrusion (ortho-amenable)	Severity	3 (2–3)	3 (3–4)	1062	-2.70	0.004*	0.069	0.26	-1.00, 0.00
	Prominence	4 (3–4)	4 (3–4)	1556	0.26	0.782	1.000	0.02	-1.00, 1.00
	Esthetics	2 (2–3)	2 (2–3)	1513	0.00	1.000	1.000	0.00	-1.00, 1.00
	Recommendation	2 (2–2)	3 (2–3)	831	-4.07	< 0.001*	< 0.001*	0.39	-1.00, -1.00
C4: Marked lip fullness despite satisfactory alignment	Severity	4 (4–4)	4 (3–4)	1686	1.04	0.254	1.000	0.10	0.00, 0.00
	Prominence	4 (3–4)	4 (4–4)	1138	-2.24	0.016*	0.272	0.21	-1.00, 0.00
	Esthetics	2 (2–3)	2 (2–3)	1658	0.87	0.356	1.000	0.08	0.00, 1.00
	Recommendation	3 (3–4)	4 (3–5)	710	-4.79	< 0.001*	< 0.001*	0.46	-2.00, -1.00
C5: Prominent lips with incompetence and strain	Severity	4 (4–5)	4 (4–5)	1448	-0.39	0.673	1.000	0.04	0.00, 0.00
	Prominence	4 (4–5)	4 (4–5)	1652	0.84	0.367	1.000	0.08	0.00, 1.00
	Esthetics	2 (2–2)	2 (2–3)	1474	-0.23	0.803	1.000	0.02	0.00, 0.00
	Recommendation	4 (3–4)	4 (4–5)	758	-4.51	< 0.001*	< 0.001*	0.43	-2.00, 0.00
C6: Persistent lip fullness after completed orthodontic treatment	Severity	4 (3–4)	4 (3–5)	1358	-0.92	0.327	1.000	0.09	0.00, 0.00
	Prominence	4 (3–5)	4 (4–4)	1574	0.37	0.693	1.000	0.04	0.00, 0.00
	Esthetics	2 (1–2)	2 (2–3)	1231	-1.68	0.075	1.000	0.16	-1.00, 0.00
	Recommendation	4 (3–5)	4 (4–5)	1264	-1.49	0.113	1.000	0.14	-1.00, 0.00

Although the principal factor selected varied across scenarios, the overall distributions of the selected factors did not differ significantly between orthodontists and oral and maxillofacial surgeons in any scenario (all p > 0.05). Patient cosmetic concerns were the most frequently selected factors in C1, bimaxillary protrusion in C3, lip volume/fullness in C4 and C6, and lip incompetence in C5 (Table 4).

**Table 4:** Principal factors influencing recommendations across the standardized clinical scenarios in section C. Data are presented as frequency and percentage. Participants selected one principal factor from 10 predefined options for each scenario; between-group comparisons were performed using the chi-square (χ²) test of independence; Cramer's V is reported as the effect size; p < 0.05 was considered statistically significant.

Scenario	Modal factor — Orthodontists (n = 55)		Modal factor — Oral surgeons (n = 55)		Test statistics, df	p-value	Effect size (Cramer's V)
	Principal factor	N (%)	Principal factor	N (%)			
C1: Mild lip fullness, no dental protrusion	Patient's cosmetic concern	30 (55%)	Patient's cosmetic concern	22 (40%)	χ² = 11.71, df = 9	0.230	0.33
C2: Moderate lip fullness, satisfactory occlusion	Patient's cosmetic concern	16 (29%)	Lip volume/fullness	22 (40%)	χ² = 12.97, df = 9	0.164	0.34
C3: Prominent lips with bimaxillary protrusion (ortho-amenable)	Bimaxillary protrusion	19 (35%)	Bimaxillary protrusion	13 (24%)	χ² = 9.79, df = 9	0.368	0.30
C4: Marked lip fullness despite satisfactory alignment	Lip volume/fullness	16 (29%)	Lip volume/fullness	23 (42%)	χ² = 7.58, df = 9	0.577	0.26
C5: Prominent lips with incompetence and strain	Lip incompetence	15 (27%)	Lip incompetence	14 (25%)	χ² = 11.89, df = 9	0.220	0.33
C6: Persistent lip fullness after completing orthodontic treatment	Lip volume/fullness	27 (49%)	Lip volume/fullness	33 (60%)	χ² = 11.37, df = 9	0.251	0.32

Significant differences were observed in several treatment preferences and decision-making measures. The groups differed in their deferral of cosmetic reduction when orthodontic treatment was expected to reduce lip prominence (p < 0.001), recommendation of reduction when fullness persisted after alignment (p < 0.001), referral practices (p = 0.026), and preference for multidisciplinary decision-making (p < 0.001). The initial treatment choice for dental protrusion with prominent lips did not differ significantly between groups (p = 0.865) (Table 5).

**Table 5:** Treatment preferences and clinical decision-making in section D. Data are presented as frequency and percentage; between-group comparisons were performed using the chi-square (χ²) test of independence. Cramer's V is reported as the effect size for contingency tables; *p < 0.05 was considered statistically significant.

Item	Orthodontists (n = 55)		Oral surgeons (n = 55)		Test statistics, df	p-value	Effect size (Cramer's V)
	Modal response	N (%)	Modal response	N (%)			
D1. Initial treatment choice for dental protrusion with prominent lips	Depends on severity	20 (36%)	Depends on severity	19 (35%)	χ² = 0.74, df = 4	0.865	0.08
D2. Would defer cosmetic reduction if orthodontics reduces prominence	Definitely yes	22 (40%)	Probably yes	15 (27%)	χ² = 22.97, df = 4	< 0.001*	0.46
D3. Would recommend reduction if fullness persists post-alignment	Probably yes	18 (33%)	Definitely yes	21 (38%)	χ² = 21.87, df = 4	< 0.001*	0.45
D4. Primary characterization of cosmetic lip reduction	Appropriate only in selected patients	19 (35%)	Appropriate only in selected patients	18 (33%)	χ² = 19.15, df = 4	< 0.001*	0.42
D9. Refer for another specialist's opinion before recommending	Usually	19 (35%)	Sometimes	21 (38%)	χ² = 11.01, df = 4	0.026*	0.32
D10. Preferred specialist for multidisciplinary decision-making	Multidisciplinary team	19 (35%)	Multidisciplinary team	24 (44%)	χ² = 33.76, df = 4	< 0.001*	0.55

Oral and maxillofacial surgeons assigned greater importance to objective facial analysis (p < 0.001) and reported greater confidence in making recommendations (p = 0.009), whereas orthodontists placed greater importance on orthodontic correction before considering surgery (p < 0.001). The perceived importance of the patient's subjective perception did not differ significantly between the groups (p = 0.069) (Table 6).

**Table 6:** Importance and confidence ratings in clinical decision-making in section D. Data are presented as median (interquartile range as IQR); items were rated on a 1–5 scale and compared between groups using the Mann–Whitney U test; effect size is reported as r; Holm-adjusted p-values were calculated across the four ordinal items to control the family-wise error rate; *p < 0.05 was considered statistically significant after Holm adjustment.

Item	Orthodontists (n = 55), median (IQR)	Oral surgeons (n = 55), median (IQR)	U value	Z value	p-value (Holm-adjusted)	r	95% CI median difference
D5. Importance of patient's subjective perception of lip fullness	4 (3–4)	4 (3–4)	1816	1.82	0.069	0.17	0.00, 1.00
D6. Importance of objective facial analysis	3 (3–4)	4 (4–5)	890	-3.72	< 0.001*	0.35	-1.00, 0.00
D7. Importance of orthodontic correction before considering surgery	4 (4–5)	3 (3–4)	2292	4.66	< 0.001*	0.44	0.00, 1.03
D8. Confidence in making a recommendation	3 (3–4)	4 (3–5)	1061	-2.70	0.009*	0.26	-1.00, 0.00

The questionnaire demonstrated good internal consistency, with Cronbach's α values of 0.85 for Section B, 0.88 for Section C, and 0.79 for Section D (Table 7).

**Table 7:** Internal consistency of the questionnaire domains (Cronbach’s alpha). Data are presented as Cronbach's alpha coefficients with 95% confidence intervals (CI); higher α values indicate greater internal consistency.

Domain	Number of items	Cronbach's α	95% CI	Interpretation
Section B: Clinical factors	20	0.85	0.80-0.89	Good
Section C: Case assessment	6	0.88	0.84-0.92	Good
Section D: Treatment preferences	10	0.79	0.68-0.88	Good

## Discussion

The present study demonstrated clear differences between orthodontists and oral and maxillofacial surgeons in their approach to cosmetic lip reduction. Oral and maxillofacial surgeons assigned greater importance to lip-centric factors, whereas orthodontists placed greater emphasis on dental/skeletal and patient-related factors. The groups also differed in their recommendations for cosmetic lip reduction across five of the six standardized clinical scenarios, with oral and maxillofacial surgeons generally demonstrating a greater tendency to recommend the reduction. This pattern is consistent with previous research showing that orthodontists and oral surgeons may share a broad agreement in facial profile assessment while differing in their preferred treatment approaches [10,11]. Bell et al. [10] reported that orthodontists and oral surgeons evaluated facial profiles similarly, but oral surgeons were more likely to recommend surgical correction. Similarly, Soh et al. [11] found a strong correlation between orthodontists' and oral surgeons' assessments of facial attractiveness, although differences in the preferred profiles were observed between the professional groups.

The greater emphasis placed on lip-centric characteristics by oral and maxillofacial surgeons is clinically understandable. Lip fullness, overall lip volume, and lip projection are direct soft-tissue characteristics that can be surgically addressed when they remain excessive despite satisfactory dental and skeletal relationships [6]. Reduction cheiloplasty is specifically intended to decrease excessive lip volume and projection and restore an appropriate relationship between the upper and lower lips [12]. Niamtu [6] described cosmetic lip reduction as a procedure directed toward enlarged lips, emphasizing appropriate patient selection and diagnostic assessment, whereas Fanous et al. [13] highlighted the importance of reducing lip volume while preserving an esthetically appropriate lip contour and upper-to-lower lip relationship.

In contrast, orthodontists assigned substantially greater importance to dental and skeletal factors. This finding is consistent with the fundamental role of orthodontic diagnosis in evaluating the relationship between tooth position, skeletal morphology, and overlying soft tissues [14]. Apparent lip prominence does not necessarily represent excessive lip volume; it may reflect incisor proclination, bimaxillary dental protrusion, increased overjet, or broader facial profile relationships [5]. Previous studies have emphasized that facial harmony should be assessed through the interaction of dental, skeletal, and soft-tissue structures rather than through isolated cephalometric or dental measurements [15]. In addition, the response of the lips to changes in incisor position is not uniform among individuals. A study using simulated maxillary incisor protraction demonstrated that the upper lip advanced by approximately 50% of the applied displacement, with lip tone and elasticity significantly influencing the magnitude of lip displacement [16]. These findings support the importance of considering individual soft tissue characteristics when anticipating changes in lip prominence following orthodontic treatment.

The importance assigned to orthodontic factors is particularly relevant to scenarios involving bimaxillary protrusion and the expected response to orthodontic treatment. In the present study, bimaxillary protrusion was the most frequently selected principal factor in the third scenario for both groups, although the overall distribution of principal factors did not differ significantly. Orthodontic treatment can modify the soft-tissue profile when the incisor position is altered. A systematic review and meta-analysis reported that extraction-based orthodontic treatment was associated with upper- and lower-lip retraction and an increase in the nasolabial angle, although the authors emphasized substantial heterogeneity and very low certainty of evidence [17]. More broadly, systematic evidence indicates that orthodontic treatment can improve facial attractiveness, although the magnitude of improvement is generally modest [18]. Thus, the greater importance that orthodontists assigned to dental/skeletal factors and orthodontic correction before surgery in the present study may reflect their greater emphasis on addressing a potentially modifiable dentofacial cause before undertaking irreversible soft-tissue surgery.

The scenario-based findings provide an important refinement of this interpretation. Oral and maxillofacial surgeons gave significantly higher recommendations for cosmetic lip reduction in the first five scenarios, including scenarios involving mild or moderate lip fullness, bimaxillary protrusion, marked fullness despite satisfactory alignment, and lip incompetence with strain. However, no significant difference was observed when lip fullness persisted after completing orthodontic treatment. This latter finding suggests a degree of convergence between the specialties once the potential orthodontic component has already been addressed. The absence of a significant difference in the sixth scenario is particularly relevant because persistent soft-tissue fullness after completion of orthodontic treatment represents a clinical situation in which an underlying dentoalveolar explanation is less likely to account for the residual appearance. This interpretation is compatible with the established principles of reduction cheiloplasty, in which persistent excessive lip volume or projection may be considered when the relevant underlying conditions have been appropriately evaluated [6,18].

The treatment preference findings further support the difference in sequencing between specialties. Orthodontists placed greater importance on orthodontic correction before considering surgery, whereas oral and maxillofacial surgeons assigned greater importance to objective facial analysis and reported greater confidence in making recommendations. The groups also differed significantly in their responses regarding the deferral of cosmetic reduction when orthodontic treatment was expected to reduce lip prominence and the recommendation of reduction when fullness persisted after alignment. These findings suggest that the difference between the specialties is not simply a difference in whether lip reduction is considered but also in when it is considered in the treatment sequence. Previous studies on professional facial profile perception have similarly demonstrated that specialist background can influence judgments of facial form and surgical need [19,20].

Patient perception remains important in this context. In the present study, patient-related factors were rated more highly by orthodontists, while the specific importance assigned to subjective perception of lip fullness did not differ significantly between the groups. This finding is clinically relevant because esthetic treatment decisions are inherently influenced by the patient's perception of the perceived deformity and desired outcome [21]. Previous work has shown that patients' perceptions of their own facial profiles may differ from those of clinicians and that self-perception can influence the willingness to undergo corrective surgery [10]. The contemporary literature also emphasizes that lip attractiveness is influenced by lip volume, proportion, facial context, age, sex, and cultural background, indicating that a universally ideal lip size should not be assumed [22].

The principal-factor analysis provided another important finding. Although the modal factor varied between scenarios, for example, patient cosmetic concern was most frequently selected in the mild-fullness scenario, bimaxillary protrusion in the orthodontically amenable protrusion scenario, and lip volume/fullness in scenarios involving marked or persistent fullness, the complete distributions of the 10 possible factors did not differ significantly between orthodontists and oral and maxillofacial surgeons. Therefore, the specialties appear to share a broadly similar range of considerations, even though they differ in the relative importance assigned to specific clinical domains and in their final treatment recommendations. This distinction is important because a difference in treatment recommendations should not be interpreted as evidence that the two groups use fundamentally different diagnostic principles.

The findings also have implications for multidisciplinary treatment planning. Both groups demonstrated a preference for multidisciplinary involvement, although the distribution differed significantly, with oral and maxillofacial surgeons more frequently selecting a multidisciplinary team. This is consistent with the multifactorial nature of lip and facial esthetics, in which soft-tissue morphology, dental position, skeletal relationships, facial proportions, smile characteristics, and patient expectations interact. Previous literature similarly emphasizes the importance of evaluating lips in relation to the surrounding facial structures and recognizing that harmonious facial aesthetics cannot be reduced to dental occlusion alone [23]. Therefore, a coordinated orthodontic and surgical assessment may be particularly valuable when the cause of apparent lip prominence is uncertain or when both dentoalveolar and soft-tissue components are present.

The distinction between cosmetic lip reduction and lip-lift procedures should also be maintained. Reduction cheiloplasty primarily addresses excessive lip volume or projection, whereas a subnasal lip lift is designed principally to shorten an elongated cutaneous upper lip and increase vermilion and/or tooth show [24]. Studies of subnasal lip lifting have demonstrated measurable reductions in upper-lip length and increases in vermilion display, supporting a different anatomical objective from volume-reduction cheiloplasty [24,25]. Consequently, these procedures should not be considered interchangeable, and treatment selection should be based on the specific anatomical characteristics responsible for the patient's concerns.

This study has several limitations. First, its cross-sectional questionnaire design assessed professional judgment rather than actual treatment outcomes. Second, standardized written scenarios ensured consistency between participants but could not reproduce the complexity of an in-person clinical examination, including dynamic lip movement, three-dimensional facial assessment, palpation, or detailed patient-clinician communication. Third, the study was conducted in a single institutional setting, which may limit generalizability to clinicians from other regions and healthcare environments. Fourth, although the questionnaire demonstrated good internal consistency across sections, Cronbach’s alpha for the treatment-preference domain was lower than that of the other sections, suggesting that these items may represent related but distinct aspects of clinical decision-making. Finally, patient-reported treatment preferences, treatment acceptance, postoperative satisfaction, and objective clinical outcomes were not assessed. These limitations mean that the observed differences should be interpreted as differences in professional decision-making rather than as evidence that one specialty’s recommendation produces superior clinical outcomes.

Future studies should recruit larger multicenter samples involving orthodontists and oral and maxillofacial surgeons from diverse geographic and professional settings. Prospective studies incorporating standardized clinical photographs, three-dimensional facial measurements, and patient-reported outcomes could determine whether the differences observed in professional recommendations translate into differences in treatment selection, satisfaction, or postoperative outcomes. Further research should also examine how much orthodontic correction changes perceived lip prominence and whether the persistence of soft-tissue fullness after orthodontic treatment influences subsequent acceptance of cosmetic lip reduction. The development of multidisciplinary assessment protocols that integrate dental/skeletal analysis, lip morphology, facial esthetics, patient perception, and expected orthodontic response may ultimately help clinicians select appropriate candidates for cosmetic lip procedures while minimizing unnecessary irreversible interventions.

## Conclusions

The present study demonstrated that orthodontists and oral and maxillofacial surgeons differed in their recommendations for cosmetic lip reduction, with oral and maxillofacial surgeons showing a greater tendency to recommend the procedure in five of the six standardized clinical scenarios. Lip-centric characteristics, particularly lip fullness and overall lip volume, were more important to surgeons, whereas orthodontists placed greater emphasis on dental and skeletal factors and orthodontic correction before considering surgery. These findings highlight the importance of distinguishing excessive soft-tissue volume from dentofacial causes of lip prominence and support an individualized, multidisciplinary approach before undertaking irreversible cosmetic intervention.

## Appendices

Study questionnaire  

**Table 8:** Study questionnaire

QUESTIONNAIRE: Clinical factors influencing recommendations for cosmetic lip reduction			
Participant information and informed consent			
☐	I have read and understood the participant information and voluntarily agree to participate.		
☐	I agree that my anonymized responses may be used for research and scientific publication.		
Section A. Demographic and professional characteristics			
No.	Question	Response	
A1	Age	_____ years	
A2	Sex	☐ Male ☐ Female	
A3	Professional specialty	☐ Orthodontist ☐ Oral and maxillofacial surgeon	
A4	Highest professional qualification	☐ MDS ☐ DNB ☐ Other: __________	
A5	Years of independent clinical experience	☐ 1–5 years ☐ 6–10 years ☐ >10 years	
A6	Current practice setting	☐ Academic institution ☐ Hospital-based practice ☐ Private practice ☐ Other: __________	
A7	Do you have previous experience with cosmetic lip procedures?	☐ Yes ☐ No	
A8	Approximately how many patients with cosmetic lip concerns have you personally evaluated?	☐ None ☐ 1–10 ☐ 11–25 ☐ 26–50 ☐ >50	
Section B. Importance of clinical factors: Please indicate how important each factor is when deciding whether cosmetic lip reduction should be recommended.			
No.	Clinical factor	Response	
B1	Degree of upper lip fullness	☐ 1 ☐ 2 ☐ 3 ☐ 4 ☐ 5	
B2	Degree of lower lip fullness	☐ 1 ☐ 2 ☐ 3 ☐ 4 ☐ 5	
B3	Overall lip volume	☐ 1 ☐ 2 ☐ 3 ☐ 4 ☐ 5	
B4	Lip protrusion	☐ 1 ☐ 2 ☐ 3 ☐ 4 ☐ 5	
B5	Lip incompetence	☐ 1 ☐ 2 ☐ 3 ☐ 4 ☐ 5	
B6	Lip strain on closure	☐ 1 ☐ 2 ☐ 3 ☐ 4 ☐ 5	
B7	Vermilion show	☐ 1 ☐ 2 ☐ 3 ☐ 4 ☐ 5	
B8	Lip asymmetry	☐ 1 ☐ 2 ☐ 3 ☐ 4 ☐ 5	
B9	Gingival display	☐ 1 ☐ 2 ☐ 3 ☐ 4 ☐ 5	
B10	Smile aesthetics	☐ 1 ☐ 2 ☐ 3 ☐ 4 ☐ 5	
B11	Maxillary incisor proclination	☐ 1 ☐ 2 ☐ 3 ☐ 4 ☐ 5	
B12	Mandibular incisor proclination	☐ 1 ☐ 2 ☐ 3 ☐ 4 ☐ 5	
B13	Bimaxillary dental protrusion	☐ 1 ☐ 2 ☐ 3 ☐ 4 ☐ 5	
B14	Increased overjet	☐ 1 ☐ 2 ☐ 3 ☐ 4 ☐ 5	
B15	Facial convexity	☐ 1 ☐ 2 ☐ 3 ☐ 4 ☐ 5	
B16	Nasolabial angle	☐ 1 ☐ 2 ☐ 3 ☐ 4 ☐ 5	
B17	Chin prominence	☐ 1 ☐ 2 ☐ 3 ☐ 4 ☐ 5	
B18	Lower facial height	☐ 1 ☐ 2 ☐ 3 ☐ 4 ☐ 5	
B19	Patient's subjective cosmetic concern	☐ 1 ☐ 2 ☐ 3 ☐ 4 ☐ 5	
B20	Expected response to orthodontic treatment	☐ 1 ☐ 2 ☐ 3 ☐ 4 ☐ 5	
Response scale: 1 = Not important; 2 = Slightly important; 3 = Moderately important; 4 = Very important; 5 = Extremely important.			
Section C. Standardized written clinical scenarios		Assessment	Response
C1	Case 1 A patient presents with mild upper lip fullness and mild overall lip prominence. Lip closure is competent without visible strain. There is no clinically significant dental protrusion, incisor proclination, or major facial-profile discrepancy. The patient's cosmetic concern is limited.	Severity of lip fullness	☐ 1 ☐ 2 ☐ 3 ☐ 4 ☐ 5
		Overall lip prominence	☐ 1 ☐ 2 ☐ 3 ☐ 4 ☐ 5
		Overall facial aesthetics	☐ 1 ☐ 2 ☐ 3 ☐ 4 ☐ 5
		Recommendation for cosmetic lip reduction	☐ 1 ☐ 2 ☐ 3 ☐ 4 ☐ 5
	Select principal factor: ☐ Lip volume/fullness, ☐ Lip incompetence, ☐ Incisor proclination, ☐ Facial profile. ☐ Smile aesthetics, ☐ Lip protrusion, ☐ Lip strain, ☐ Bimaxillary protrusion, ☐ Gingival display, ☐ Patient's cosmetic concern		
C2	Case 2 A patient presents with moderate upper and lower lip fullness and increased overall lip volume. Lip closure is competent, with no significant strain. Dental alignment is satisfactory, and there is no marked bimaxillary protrusion. The patient reports a clear cosmetic concern regarding excessive lip fullness.	Severity of lip fullness	☐ 1 ☐ 2 ☐ 3 ☐ 4 ☐ 5
		Overall lip prominence	☐ 1 ☐ 2 ☐ 3 ☐ 4 ☐ 5
		Overall facial aesthetics	☐ 1 ☐ 2 ☐ 3 ☐ 4 ☐ 5
		Recommendation for cosmetic lip reduction	☐ 1 ☐ 2 ☐ 3 ☐ 4 ☐ 5
	Select principal factor: ☐ Lip volume/fullness, ☐ Lip incompetence, ☐ Incisor proclination, ☐ Facial profile. ☐ Smile aesthetics, ☐ Lip protrusion, ☐ Lip strain, ☐ Bimaxillary protrusion, ☐ Gingival display, ☐ Patient's cosmetic concern		
C3	Case 3 A patient presents with prominent lips and moderate bimaxillary dental protrusion with proclined incisors and increased overjet. Lip fullness is mild to moderate. The facial profile is convex, and orthodontic treatment is expected to reduce lip prominence.	Severity of lip fullness	☐ 1 ☐ 2 ☐ 3 ☐ 4 ☐ 5
		Overall lip prominence	☐ 1 ☐ 2 ☐ 3 ☐ 4 ☐ 5
		Overall facial aesthetics	☐ 1 ☐ 2 ☐ 3 ☐ 4 ☐ 5
		Recommendation for cosmetic lip reduction	☐ 1 ☐ 2 ☐ 3 ☐ 4 ☐ 5
	Select principal factor: ☐ Lip volume/fullness, ☐ Lip incompetence, ☐ Incisor proclination, ☐ Facial profile. ☐ Smile aesthetics, ☐ Lip protrusion, ☐ Lip strain, ☐ Bimaxillary protrusion, ☐ Gingival display, ☐ Patient's cosmetic concern		
C4	Case 4 A patient presents with marked upper and lower lip fullness and increased lip volume despite satisfactory dental alignment. There is no significant incisor proclination or bimaxillary dental protrusion. Lip closure is competent, and the principal complaint is excessive lip fullness.	Severity of lip fullness	☐ 1 ☐ 2 ☐ 3 ☐ 4 ☐ 5
		Overall lip prominence	☐ 1 ☐ 2 ☐ 3 ☐ 4 ☐ 5
		Overall facial aesthetics	☐ 1 ☐ 2 ☐ 3 ☐ 4 ☐ 5
		Recommendation for cosmetic lip reduction	☐ 1 ☐ 2 ☐ 3 ☐ 4 ☐ 5
	☐ Lip volume/fullness, ☐ Lip incompetence, ☐ Incisor proclination, ☐ Facial profile. ☐ Smile aesthetics, ☐ Lip protrusion, ☐ Lip strain, ☐ Bimaxillary protrusion, ☐ Gingival display, ☐ Patient's cosmetic concern		
C5	Case 5 A patient presents with prominent lips, lip incompetence, and visible lip strain on attempted closure. There is moderate dental protrusion and a convex facial profile. The patient is concerned about both facial appearance and difficulty achieving comfortable lip closure.	Severity of lip fullness	☐ 1 ☐ 2 ☐ 3 ☐ 4 ☐ 5
		Overall lip prominence	☐ 1 ☐ 2 ☐ 3 ☐ 4 ☐ 5
		Overall facial aesthetics	☐ 1 ☐ 2 ☐ 3 ☐ 4 ☐ 5
		Recommendation for cosmetic lip reduction	☐ 1 ☐ 2 ☐ 3 ☐ 4 ☐ 5
	Select principal factor: ☐ Lip volume/fullness, ☐ Lip incompetence, ☐ Incisor proclination, ☐ Facial profile. ☐ Smile aesthetics, ☐ Lip protrusion, ☐ Lip strain, ☐ Bimaxillary protrusion, ☐ Gingival display, ☐ Patient's cosmetic concern		
C6	Case 6 A patient has completed orthodontic treatment with satisfactory dental alignment and substantial correction of dental protrusion. Despite correction, excessive upper and lower lip fullness and persistent lip prominence remain. The patient continues to express a strong cosmetic concern about lip volume	Severity of lip fullness	☐ 1 ☐ 2 ☐ 3 ☐ 4 ☐ 5
		Overall lip prominence	☐ 1 ☐ 2 ☐ 3 ☐ 4 ☐ 5
		Overall facial aesthetics	☐ 1 ☐ 2 ☐ 3 ☐ 4 ☐ 5
		Recommendation for cosmetic lip reduction	☐ 1 ☐ 2 ☐ 3 ☐ 4 ☐ 5
	Select principal factor: ☐ Lip volume/fullness, ☐ Lip incompetence, ☐ Incisor proclination, ☐ Facial profile. ☐ Smile aesthetics, ☐ Lip protrusion, ☐ Lip strain, ☐ Bimaxillary protrusion, ☐ Gingival display, ☐ Patient's cosmetic concern		
For severity: 1 = Very mild; 2 = Mild; 3 = Moderate; 4 = Marked; 5 = Severe. For facial aesthetics: 1 = Very unattractive; 2 = Unattractive; 3 = Neutral/acceptable; 4 = Attractive; 5 = Very attractive. For recommendation: 1 = Definitely not recommended; 2 = Probably not recommended; 3 = Uncertain; 4 = Probably recommended; 5 = Definitely recommended.			
Section D. Treatment preference and clinical decision-making			
No.	Question	Response	
D1	If a patient has significant dental protrusion and prominent lips, which treatment would you consider first?	☐ Orthodontic treatment ☐ Cosmetic lip reduction ☐ Orthodontic treatment + cosmetic lip reduction ☐ Depends on severity ☐ Neither	
D2	If orthodontic treatment is expected to reduce lip prominence substantially, would you defer cosmetic lip reduction?	☐ Definitely yes ☐ Probably yes ☐ Uncertain ☐ Probably no ☐ Definitely no	
D3	If satisfactory dental alignment is achieved but excessive lip fullness persists, would you recommend cosmetic lip reduction?	☐ Definitely yes ☐ Probably yes ☐ Uncertain ☐ Probably no ☐ Definitely no	
D4	In your opinion, cosmetic lip reduction is primarily:	☐ A standalone aesthetic procedure ☐ An adjunct to orthodontic treatment ☐ An alternative when orthodontic treatment cannot adequately improve lip aesthetics ☐ Appropriate only in selected patients ☐ Generally unnecessary when an orthodontic/dentoalveolar cause is present	
D5	How important is the patient's subjective perception of lip fullness in your decision?	☐ 1 ☐ 2 ☐ 3 ☐ 4 ☐ 5	
D6	How important is objective facial analysis in your decision?	☐ 1 ☐ 2 ☐ 3 ☐ 4 ☐ 5	
D7	How important is orthodontic correction before considering cosmetic lip surgery?	☐ 1 ☐ 2 ☐ 3 ☐ 4 ☐ 5	
D8	How confident are you in making a recommendation regarding cosmetic lip reduction?	☐ 1 ☐ 2 ☐ 3 ☐ 4 ☐ 5	
D9	Would you refer a patient for another specialist's opinion before recommending cosmetic lip reduction?	☐ Always ☐ Usually ☐ Sometimes ☐ Rarely ☐ Never	
D10	Which specialist would you preferentially involve in decision-making for a patient with prominent lips and dental protrusion?	☐ Orthodontist ☐ Oral and maxillofacial surgeon ☐ Plastic/aesthetic surgeon ☐ Multidisciplinary team ☐ Depends on the clinical presentation	
For D5 and D6: 1 = Not important; 2 = Slightly important; 3 = Moderately important; 4 = Very important; 5 = Extremely important. For D7: the same 5-point importance scale applies. For D8: 1 = Very low confidence; 2 = Low confidence; 3 = Moderate confidence; 4 = High confidence; 5 = Very high confidence.			
